# THE EXPERIENCE OF THE COVID-19 PANDEMIC AMONG OLDER ADULTS IN PUERTO RICO

**DOI:** 10.1093/geroni/igac059.618

**Published:** 2022-12-20

**Authors:** Denise Burnette, Yamile Martí Haidar

## Abstract

Older age groups and racial and ethnic populations are more likely to have medical conditions that increase their risks for illness, hospitalization and death from COVID-19. Puerto Rico has a high prevalence of acute and chronic health conditions and the lowest percent of people reporting good / excellent health of all U.S. states and territories. The effects of poor health on the island have been compounded in recent years by economic and political instability and by major natural disasters; yet, as of February 2022, 95% of persons aged 65 and older had received at least one vaccination and 88% were fully vaccinated. This symposium comprises five papers based on a study of personal, social, physical health, and mental health factors associated with COVID-19 in a community sample of 213 adults aged 60 and over in Puerto Rico. The first paper will introduce the study, including its purpose, theoretical framework, and methodology. Next, we will address methodological and ethical challenges of conducting research with older adults during the pandemic and strategies we used to overcome these. The third paper will report on the core focus of the project: to assess the knowledge, attitudes, and practices of older Puerto Ricans toward COVID-19. The fourth paper will focus on mental health effects of loneliness and loss, two of the most challenging exigencies of the pandemic for older adults. Finally, we will consider participants’ responses to open-ended questions about how they have coped with the pandemic and made meaning of their experiences.

